# Supplementary material for: Components of the ribosome biogenesis pathway underlie establishment of telomere length set point in Arabidopsis
Source: Nat Commun. 2019 Dec 2;10:5479. doi: 10.1038/s41467-019-13448-z (PMC6889149; doi:10.1038/s41467-019-13448-z)
Supplement: Supplementary file 1 — Supplementary Information [file 41467_2019_13448_MOESM1_ESM.pdf]

Components of the ribosome biogenesis pathway underlie establishment of telomere length set point in Arabidopsis.

Abdulkina *et al.*

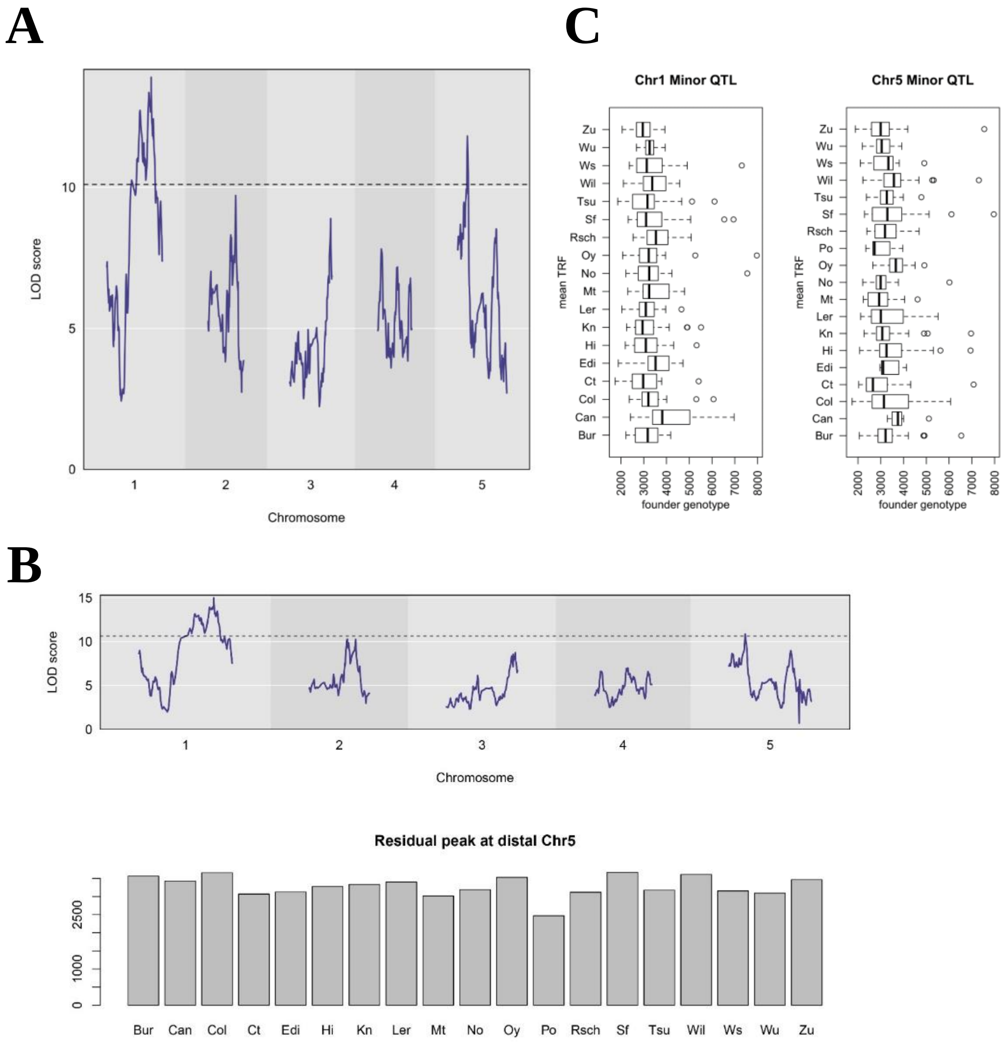

Supplementary Figure 1. Analysis of minor QTL peaks on Chr 1 and Chr 5. A, One-way QTL scan of mean telomere length in the MAGIC population controlling for the presence of the primary QTL on Chr 5 by including probabilities of the Sf-2 haplotype at the primary QTL as an additive covariate. The two minor QTL on Chr 1 and Chr 5 collectively account for 21.5% of the total telomere length variance in the MAGIC population. **B**, Conditional QTL LOD profile derived from the scan1 function of R/qtl2. The scan (upper panel) is conditioned on the genotype frequencies at the primary QTL peak. The dashed line indicates an empirical LOD threshold for GWER controlled alpha = 0.05 threshold. Lower panel depicts phenotypic means for each of the 19 parental founder genotypes. **C,** Boxplots (boxes are the interquartile range, whiskers capture all points within 1.5IQR, outlying points are observations beyond the 1.5IQR) show the contribution of 19 parental haplotypes at two minor QTL on Chr 1 and Chr 5 to telomere length (in kb) in *Arabidopsis thaliana* MAGIC lines.

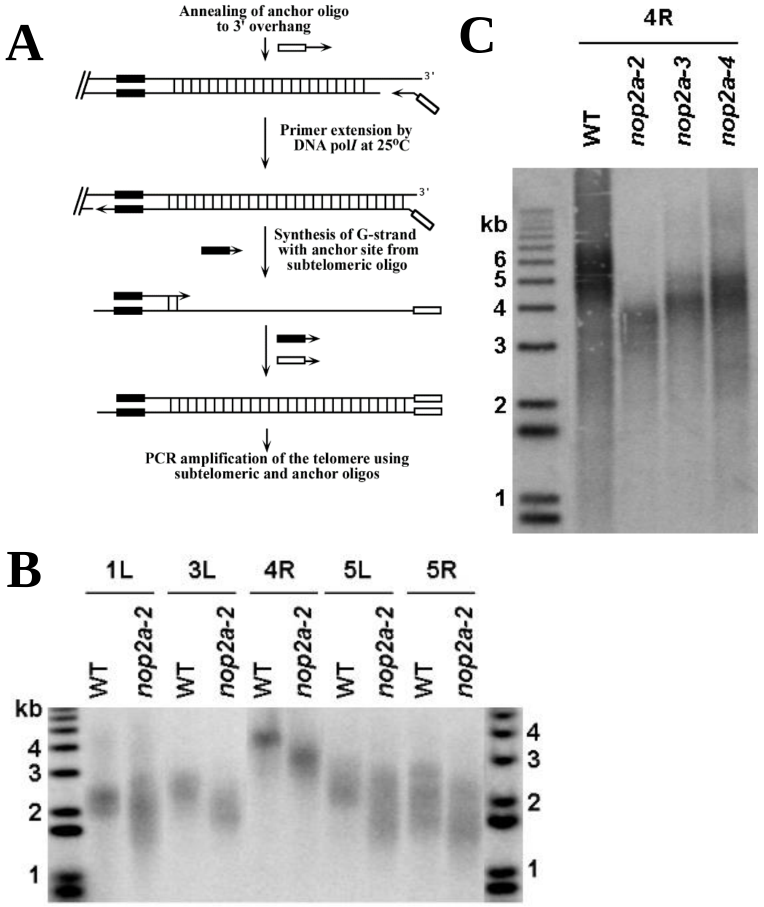

Supplementary Figure 2. Measurement of telomere length on individual Arabidopsis chromosome arms in wild type and *nop2a* mutants by PETRA. A, Diagram of the PETRA method. PETRA is a PCR-based technique that uses unique subtelomeric sequences at individual chromosome ends to accurately measure telomere length in a single plant on a single chromosome arm. **B,** PETRA for 5 different chromosome arms using wild type and *nop2a-2* DNA templates. Telomeres at individual chromosome arms are shorter in *nop2a-2* mutant compared to wild type. **C,** PETRA for right arm of chromosome 4 (4R) using wild type, *nop2a-2*, *nop2a-3* and *nop2a-4* DNA templates. 4R telomeres are shorter in all three *nop2a* mutants compared to the wild type.

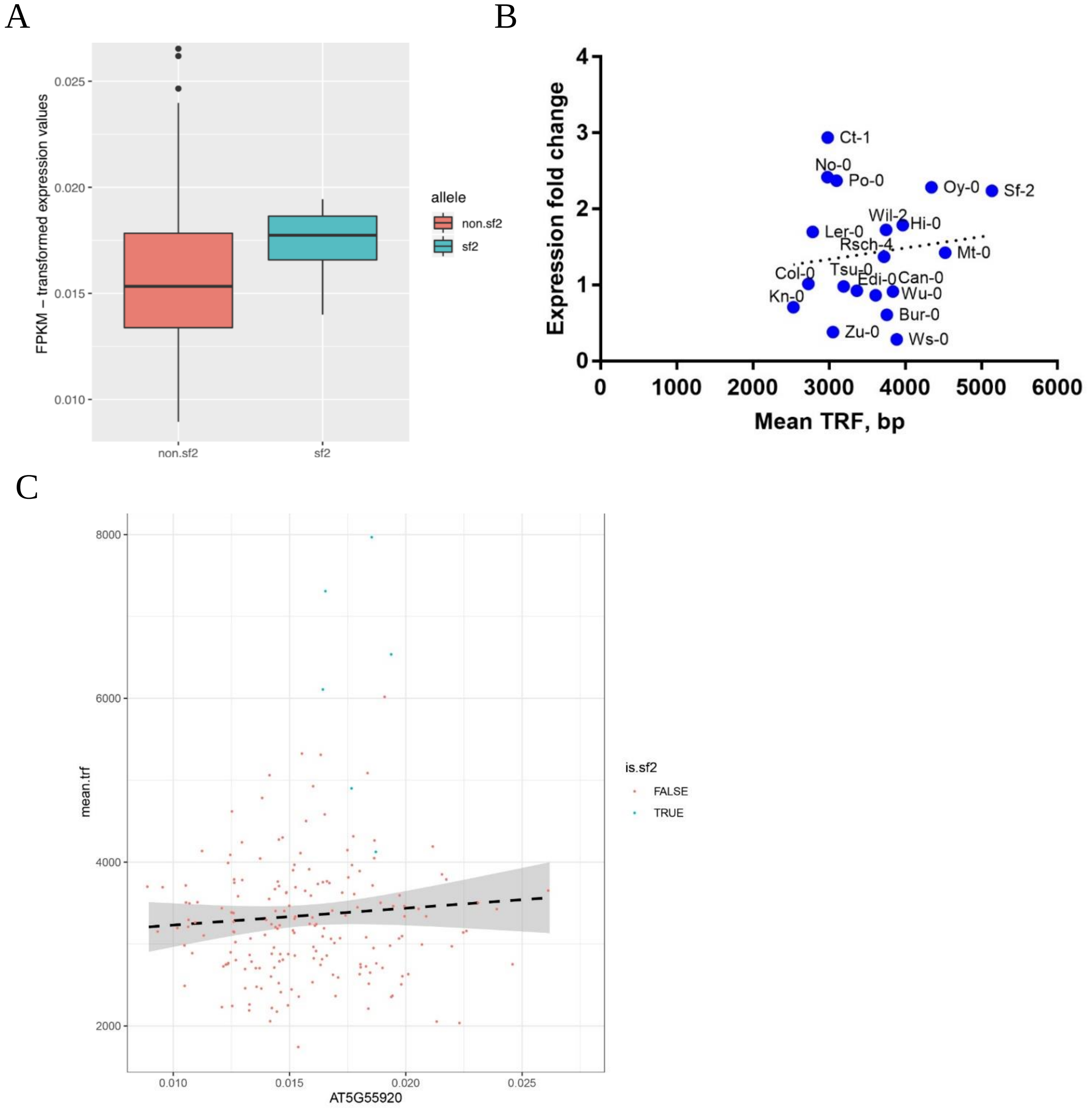

Supplementary Figure 3. Analysis of *NOP2A* expression. A, Comparison of *NOP2A* expression in 8 MAGIC lines harboring Sf-2 specific *NOP2A* allele at major effect Chr 5 QTL versus all other MAGIC lines with known *NOP2A* expression values. p-value = 0.04106, ANOVA. Error bars represent standard errors for each allele. **B**, Scatterplot of correlations between *NOP2A* gene expression in the 19 MAGIC founder genotypes and their telomere length. y = 0.0001x + 0.8931, $R^2 = 0.0169$. **C,** Mean TRF - *NOP2A* expression correlations across the MAGIC lines that were subjected to gene expression assays. Points are colored by whether the plant has an Sf-2 (blue) or non-Sf-2 (red) genotype at the *NOP2A* QTL. Linear model fit and 95% confidence is plotted as the vector and surrounding polygon. Colors in (C) follow (A).

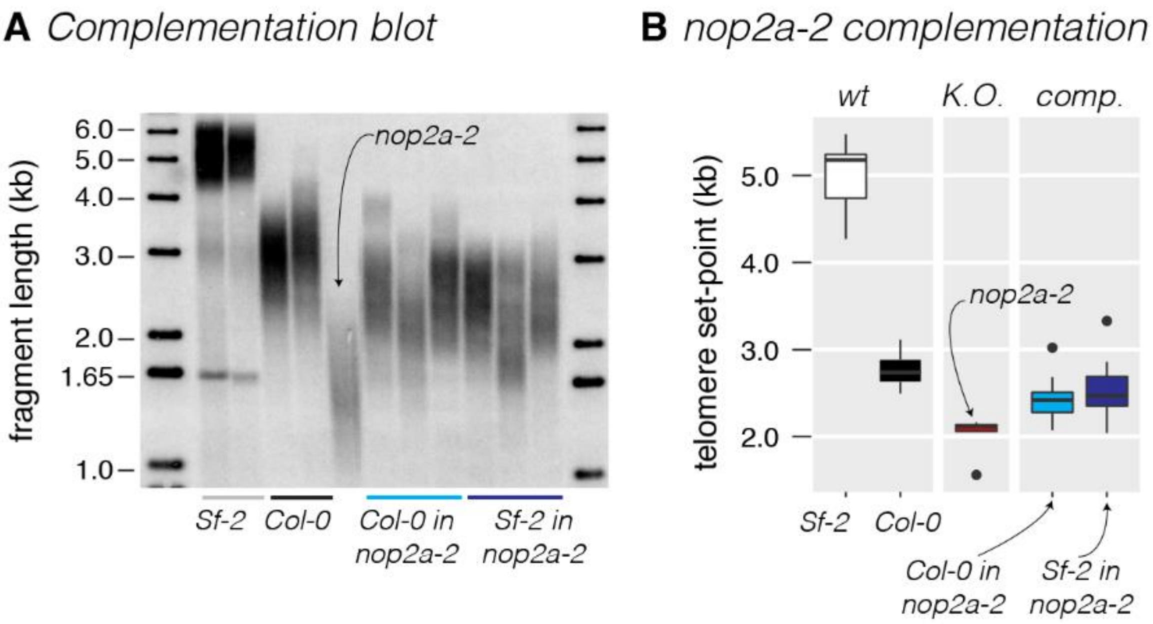

Supplementary Figure 4. Transformants expressing Col-0 and Sf-2 specific *NOP2A* variants restore telomere length of the *nop2a-2* mutants up to Col-0 wild type level. A, TRF analysis of wild type Sf-2 and Col-0 accessions, untransformed *nop2a-2* mutants and individual *nop2a-2* transformants harboring *NOP2A* alleles from Col-0 and Sf-2 accessions. All analyzed transformed plants are primary transformants (the first plant generation, T1). **B,** Quantification of complementation efficiency of *NOP2A* alleles from Col-0 and Sf-2 accessions introduced into the *nop2a-2* mutant background. N= 13 (Sf-2), 10 (Col-0), 7 (*nop2a-2*), 11 (T1 Col-0 into *nop2a-2*), 15 (Sf-2 into *nop2a-2*). Error bars represent standard errors for each wild type, mutant or independently transformed line of each genotype.

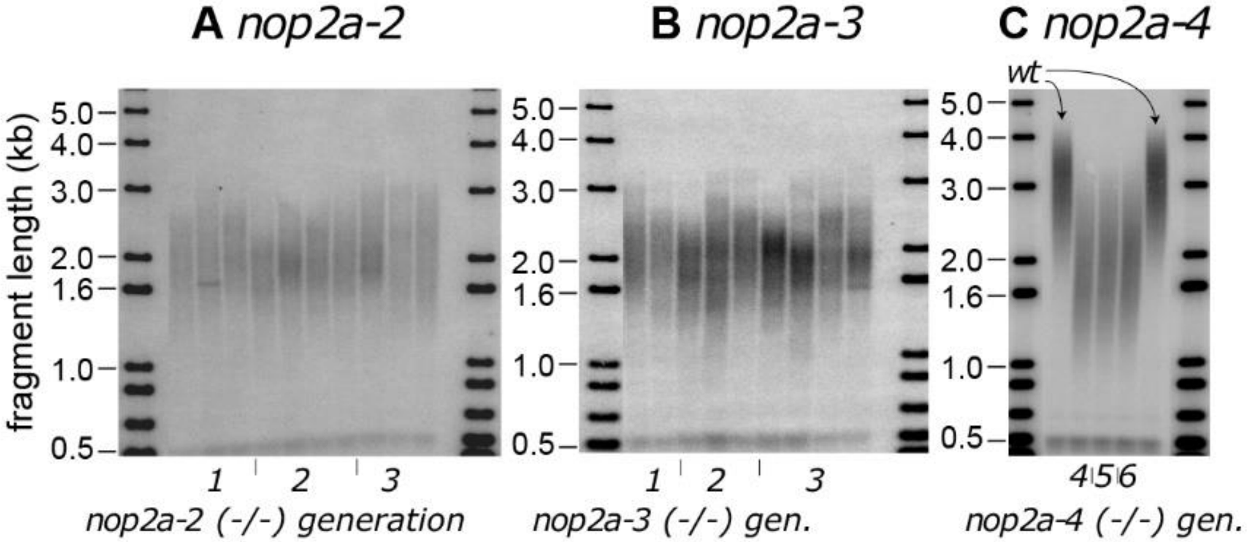

Supplementary Figure 5. Telomere length in several consecutive generations of homozygous *nop2a-2, nop2a-3* and *nop2a-4* mutants. Telomere length is stable at a new shorter set point in three generations of *nop2a-2* (**A**) and *nop2a-3* (**B**) mutant plants. Asterisks indicate generations propagated in the lab, with G1* being the original mutant *nop2a* seeds received directly from the ABRC stock center. **C**, Telomere length analysis in three late generations (G3, G4, G5) of *nop2a-4*$^{-/-}$ mutants.
