# Supplementary material for: Components of the ribosome biogenesis pathway underlie establishment of telomere length set point in Arabidopsis
Source: Nat Commun. 2019 Dec 2;10:5479. doi: 10.1038/s41467-019-13448-z (PMC6889149; doi:10.1038/s41467-019-13448-z)
Supplement: Supplementary file 3 — Description of Additional Supplementary Files [file 41467_2019_13448_MOESM3_ESM.pdf]

Description of Additional Supplementary Files

File Name: Supplementary Data 1
Description: Telomere length measurement data for 19 MAGIC founder genotypes.

File Name: Supplementary Data 2
Description: Telomere length measurement data for 480 MAGIC lines.

File Name: Supplementary Data 3
Description: List of Chr 5 QTL candidate genes with one or more Sf-2 specific DNA polymorphisms.

File Name: Supplementary Data 4
Description: List of Chr 5 QTL candidate genes with unique expression pattern in Sf-2 ecotype.

File Name: Supplementary Data 5
Description: Telomere length phenotypes of mutant plant lines with T-DNA insertions inside selected Chr 5 QTL interval genes.

File Name: Supplementary Data 6
Description: Nucleotide sequence polymorphism in the NOP2A locus in Col-0 and Sf-2 parents.

File Name: Supplementary Data 7
Description: Summary of fold changes in NOP2A transcript level in the 18 MAGIC founder genotypes over Col-0 reference genotype.
