# Supplementary material for: Components of the ribosome biogenesis pathway underlie establishment of telomere length set point in Arabidopsis
Source: Nat Commun. 2019 Dec 2;10:5479. doi: 10.1038/s41467-019-13448-z (PMC6889149; doi:10.1038/s41467-019-13448-z)
Supplement: Supplementary file 11 — Reporting Summary [file 41467_2019_13448_MOESM11_ESM.pdf]

nature research

Corresponding author(s): Dorothy E. Shippen, Thomas E. Juenger and Eugene V. Shakirov

Last updated by author(s): Nov 5, 2019

Reporting Summary

Nature Research wishes to improve the reproducibility of the work that we publish. This form provides structure for consistency and transparency in reporting. For further information on Nature Research policies, see Authors & Referees and the Editorial Policy Checklist .

Statistics

For all statistical analyses, confirm that the following items are present in the figure legend, table legend, main text, or Methods section.

n/a	Confirmed	
☐	☒	The exact sample size (*n*) for each experimental group/condition, given as a discrete number and unit of measurement
☐	☒	A statement on whether measurements were taken from distinct samples or whether the same sample was measured repeatedly
☐	☒	The statistical test(s) used AND whether they are one- or two-sided<br>*Only common tests should be described solely by name; describe more complex techniques in the Methods section.*
☐	☒	A description of all covariates tested
☐	☒	A description of any assumptions or corrections, such as tests of normality and adjustment for multiple comparisons
☐	☒	A full description of the statistical parameters including central tendency (e.g. means) or other basic estimates (e.g. regression coefficient) AND variation (e.g. standard deviation) or associated estimates of uncertainty (e.g. confidence intervals)
☐	☒	For null hypothesis testing, the test statistic (e.g. *F*, *t*, *r*) with confidence intervals, effect sizes, degrees of freedom and *P* value noted<br>*Give P values as exact values whenever suitable.*
☒	☐	For Bayesian analysis, information on the choice of priors and Markov chain Monte Carlo settings
☒	☐	For hierarchical and complex designs, identification of the appropriate level for tests and full reporting of outcomes
☐	☒	Estimates of effect sizes (e.g. Cohen's *d*, Pearson's *r*), indicating how they were calculated

Our web collection on statistics for biologists contains articles on many of the points above.

Software and code

Policy information about availability of computer code

Data collection	No software was used for data collection
Data analysis	Data for MAGIC lines were analyzed with the HAPPY package (Kover et al., 2009, PLOS Genetics)<br>QTL analysis was done in HAPPY, mpmap and R/qtl2.

For manuscripts utilizing custom algorithms or software that are central to the research but not yet described in published literature, software must be made available to editors/reviewers. We strongly encourage code deposition in a community repository (e.g. GitHub). See the Nature Research guidelines for submitting code & software for further information.

Data

Policy information about availability of data

All manuscripts must include a data availability statement. This statement should provide the following information, where applicable:

- Accession codes, unique identifiers, or web links for publicly available datasets
- A list of figures that have associated raw data
- A description of any restrictions on data availability

The authors declare that the data supporting the findings of this study are available within the paper [and its supplementary information files].

Field-specific reporting

Please select the one below that is the best fit for your research. If you are not sure, read the appropriate sections before making your selection.

☒ Life sciences ☐ Behavioural & social sciences ☐ Ecological, evolutionary & environmental sciences

Life sciences study design

All studies must disclose on these points even when the disclosure is negative.

Sample size	480 of the available Arabidopsis thaliana MAGIC lines were used in the study. For all T-DNA mutants, sample size consisted of 2-6 plants per line.
Data exclusions	No data were excluded from the analysis
Replication	To verify reproducibility, a small number of MAGIC lines were first grown in 4-6 replicates, and broad-sense heritability (H2) was found to be 0.87. Given this high degree of heritability all remaining MAGIC lines were then evaluated with 1.07 replication. For each T-DNA mutant, 2-6 individual plants per line were evaluated.
Randomization	All MAGIC lines were randomly selected from the list of available lines grown randomly in a plant growth incubator, 96 at a time. For telomere length analysis, frozen tissue was randomly selected for DNA extraction. All T-DNA mutant plants were grown randomly in a plant growth chamber together with wild type plants.
Blinding	All growing plants were assigned random numbers with specific descriptions kept separate in a xls file.

Reporting for specific materials, systems and methods

We require information from authors about some types of materials, experimental systems and methods used in many studies. Here, indicate whether each material, system or method listed is relevant to your study. If you are not sure if a list item applies to your research, read the appropriate section before selecting a response.

Materials & experimental systems

n/a	Involved in the study
☒	☐ Antibodies
☒	☐ Eukaryotic cell lines
☒	☐ Palaeontology
☒	☐ Animals and other organisms
☒	☐ Human research participants
☒	☐ Clinical data

Methods

n/a	Involved in the study
☒	☐ ChIP-seq
☒	☐ Flow cytometry
☒	☐ MRI-based neuroimaging
